# Metabolic Profiling Analysis of the Effect and Mechanism of Gushiling Capsule in Rabbits With Glucocorticoid-Induced Osteonecrosis of the Femoral Head

**DOI:** 10.3389/fphar.2022.845856

**Published:** 2022-05-02

**Authors:** Runhong Mei, Dan Chen, Duming Zhong, Guoyong Li, Shaobai Lin, Guangquan Zhang, Kaiyun Chen, Xuefeng Yu

**Affiliations:** ^1^ Department of Orthopaedics, The Fourth Affiliated Hospital of Nanchang University, Nanchang, China; ^2^ Department of Drug Clinical Trial, The Fourth Affiliated Hospital of Nanchang University, Nanchang, China

**Keywords:** Gushiling capsule, traditional chinese medicine, phospholipids metabolism, metabolomics, glucocorticoid-induced osteonecrosis of the femoral head

## Abstract

Gushiling capsule (GSLC) is an effective traditional Chinese medicine for the treatment of glucocorticoid-induced osteonecrosis of the femoral head (GIONFH). This study established the serum metabolite profiles of GSLC in rabbits and explored the metabolic mechanism and effect of GSLC on GIONFH. Seventy-five Japanese white rabbits were randomly divided into the control, model, and GSLC groups. The rabbits in the model group and the GSLC group received injection of prednisolone acetate. Meanwhile, rabbits in the GSLC group were treated by gavage at a therapeutic dose of GSLC once a day. The control group and the model group received the same volume of normal saline gavage. Three groups of serum samples were collected at different time points, and the changes in the metabolic spectrum were analyzed by ultra-high performance liquid chromatography-tandem mass spectrometry (UPLC-MS/MS). The resulting data set was analyzed using multivariate statistical analysis to identify potential biomarkers related to GSLC treatment. The metabolic pathway was analyzed by MetaboAnalyst 4.0 and a heatmap was constructed using the HEML1.0.3.7 software package. In addition, histopathological and radiography studies were carried out to verify the anti-GIONFH effects of GSLC. Principal component analysis (PCA) and partial least squares-discriminant analysis (PLS-DA) score plots revealed a significant separation trend between the control group and the model group and the GSLC group (1–3 weeks), but there were no significant differences in the GSLC group (4–6 weeks). Orthogonal PLS-DA (OPLS-DA) score plots also revealed an obvious difference between the model and the GSLC groups (4–6 weeks). Ten potential metabolite biomarkers, mainly phospholipids, were identified in rabbit serum samples and demonstrated to be associated with GIONFH. Hematoxylin and eosin staining and magnetic resonance imaging indicated that the pathological changes in femoral head necrosis in the GSLC group were less than in the model group, which was consistent with the improved serum metabolite spectrum. GSLC regulated the metabolic disorder of endogenous lipid components in GIONFH rabbits. GSLC may prevent and treat GIONFH mainly by regulating phospholipid metabolism *in vivo*.

## 1 Introduction

With the increasing clinical application of glucocorticoid (GC) therapy, the incidence of GC-induced osteonecrosis of the femoral head (GIONFH) caused by long-term or extensive use of GCs has also increased sharply. The disability rate of GIONFH is extremely high, placing a huge economic burden on society and families. In 2003, despite the success of “glucocorticoid therapy” for severe acute respiratory syndrome (SARS), which saved the lives of many patients, although also left many sequelae in patients, among which the incidence of GIONFH was as high as 53.5% (Li et al., 2004; Shen et al., 2006). Faced with the novel coronavirus disease 2019 (COVID-19) epidemic, which is similar to SARS, GC is still an important weapon in fighting infection and rescuing critically ill patients, who are therefore also at risk of developing GIONFH (Ledford, 2020; Tang et al., 2020; Chen et al., 2021). Therefore, the global medical community is interested in measures capable of preventing and treating GIONFH.

Phospholipids are structural elements of biological membranes, and the lipid bilayer (consisting mainly of phospholipids) represents an important platform for proteins involved in cell signaling, associated with apoptosis, gene expression, intercellular communication, and immune response pathways (Hu et al., 2013; Chaudhry et al., 2020). More recently, phospholipid metabolism has been shown to be associated with the initiation and/or progression of cell death. Phosphatidylserine (PS) metabolism, for example, is known to be a factor resulting in apoptosis (Segawa et al., 2014; Chaudhry et al., 2020). Wójcik et al. (Wójcik et al., 2020) also reported that phospholipid metabolism is an essential regulator of apoptosis. Currently, the pathological mechanism of GIONFH is not fully understood; however, more and more attention has been paid to apoptosis processes (Weinstein et al., 1998; Chen et al., 2020; Jing et al., 2020). An *in vivo* animal model of GIONFH was established to confirm the important role of osteocyte apoptosis in this process (Bai et al., 2016). Jin et al. (Jin et al., 2020) reported that curcumin prevents inflammatory-mediated osteocyte apoptosis in part by inhibiting the polarization of M1 macrophages through the JAK1/2-STAT1 pathway. Lysophosphatidic acid (a small phospholipid) was found to be a potent regulator of bone development and remodeling and has promising application potential in bone tissue engineering (Wu et al., 2019; Wu et al., 2020). Although great efforts have been made, the effects of phospholipid metabolism in GIONFH have been less studied.

Gushiling capsule (GSLC), a traditional Chinese herbal formula, has been widely sold to treat osteonecrosis of the femoral head (ONFH) in many hospitals and pharmacies. It is approved by the Heilongjiang Medical Products Administration (No. Z20110039), a subordinate organization of the National Medical Products Administration of China. It is composed of fifteen herbs mainly including *Salvia miltiorrhiza Bunge* [Lamiaceae], *Conioselinum anthriscoides* “*Chuanxiong*” [Apiaceae], *Panax notoginseng (Burkill) F.H.Chen* [Araliaceae] etc. GSLC is obtained by modern extraction and separation technology. There have been some reports on the regulation of lipid metabolism of the major components of GSLC, such as *Salvia miltiorrhiza Bunge* and *Conioselinum anthriscoides* “*Chuanxiong*”, which regulates lipid metabolism and improves abnormal lipid metabolism (Huang et al., 2015; Zhang et al., 2019; Yin et al., 2021). Several studies have described that disturbance in lipid metabolism are closely associated with GIONFH (Jiang et al., 2014; Song et al., 2017; Wang et al., 2021). The main functions of GSLC include “replenishing Qi and activating blood circulation, tonifying the kidney, and strengthening bone”. Clinical observations have demonstrated that GSLC exerts obvious therapeutic effects on GIONFH and its sequelae with few side effects (Xing, 2002; Zhang and Xing, 2016). The results of animal model experiments indicated that GSLC could improve lipid metabolism disorder, reduce blood lipids in the early stage, reduce the incidence of GIONFH, and alleviate the progression of the disease (Yu et al., 2014; Hongtao et al., 2015; Fan et al., 2018). However, modern research on this drug is limited and systematic investigations of its effects and mechanisms of action are scarce.

Metabolomics is a method of quantitative identification and qualitative analysis of endogenous and exogenous cellular metabolites to reveal the relative relationship of the pathway between the metabolites and the observed physiological and/or pathological changes. Metabonomics has been widely used in the detection of biomarkers, the monitoring of therapeutic effects and pathogenesis, the early detection and clinical diagnosis of tumors (Patterson et al., 2011; Zhou et al., 2012), heart disease (Chen et al., 2008; Rhee and Gerszten, 2012) and diabetes (Newgard et al., 2009; Leslie and Beyan, 2011). Recent reports have suggested that LC-MS-based metabolomics analysis could be used to investigate disease mechanism and drug effect (Jiang et al., 2009; Cheng et al., 2017; Guo et al., 2018). Especially for un-targeted metabolite analysis, ultra-high-performance liquid chromatography tandem mass spectrometry analysis (UHPLC-MS/MS) could obtain comprehensive metabolic changes, which allow the evaluation of the effect of the drug and exploration of its mechanism of action (Zhang et al., 2009; Zhao et al., 2012; Yokoi et al., 2015; Tao et al., 2017). However, no metabolomics studies on the effect and mechanism of the Gushiling capsule for the treatment of GIONFH have been reported.

In this study, a nontargeted metabolomics method based on UPLC-MS/MS was used to study the changes of serum metabolites in GIONFH rabbits and to explore the anti-GIONFH effect of GSLC and its metabolic mechanism. Potential biomarkers related to the prevention and treatment of GIONFH by GSLC were identified and their metabolic pathways discussed. Furthermore, histopathological and imaging studies were performed to investigate the anti-GIONFH effects of GSLC.

## 2 Materials and Methods

### 2.1 Chemicals

HPLC grade methanol, formic acid, and acetonitrile for MS analysis were purchased from Merck KGaA (Darmstadt, Germany). A 0.9% saline solution and a 3% pentobarbital sodium solution were obtained from the Nanchang University Central Laboratory. Gushiling capsules (No. 20110039) were obtained from the First Affiliated Hospital of Heilongjiang University of Chinese Medicine (Heilongjiang, China). The prednisolone acetate injection (Approval No. H33021520), 125 mg/5ml, was purchased from Xianju Pharmaceutical Co., Ltd. (Taizhou, China). Ultrapure water was produced using the Millipore Milli-Q Reagent Water System (Bedford, MA, United States).

### 2.2 Animal Experiments

Japanese white rabbits, half male and half female at 6 months old, body weight 2.5–3.2 kg, averaging 2.8 kg, were purchased from the Animal Experiment Center of Jiangxi University of Chinese Medicine (Nanchang, China; License Number: SYXK [gan] 2004-0001). After 1 week of adaptive feeding, the rabbits were weighed and venous blood was taken for biochemical index analysis. Except for the evaluation of anomalies in the biochemical indices, 75 rabbits were randomly divided into three groups: control group (*n* = 25), model group (*n* = 25), and GSLC group (*n* = 25). The rabbits in the model group and the GSLC group were intramuscularly injected with prednisolone acetate injection (20 mg/kg) (Wu et al., 2013; Bai et al., 2016) twice a week for 4 weeks. Meanwhile, rabbits in the GSLC group were administered gavage at a therapeutic dose of GSLC (0.3 g/kg) once a day for 6 weeks, and the control group and the model group received the same volume of normal saline gavage. Three groups of rabbit serum samples were collected at different time points (1–6 weeks). Several rabbits were executed for histopathological analysis after collecting serum samples at the corresponding time points. This study was carried out in accordance with the recommendations of the Guide for the Care and Use of Laboratory Animals of the National Institutes of Health. The protocol was approved by the Laboratory Animal Ethics Committee of the Fourth Affiliated Hospital of Nanchang University (Approval No. SFYYXLL-PJ-2021-KY002).

### 2.3 Sample Preparation

The samples were collected from rabbits at different time points. Three milliliters of blood were centrifuged at ×2,600 g for 10 min at 4°C. All serum samples were kept frozen at −80°C. Serum samples were retrieved from a −80°C refrigerator and thawed overnight in a 4°C refrigerator and then, vortexed for 3 min and 100 µl of serum was taken for metabonomic analysis. Remove the protein from the sample with 400 μl acetonitrile. The mixture was mixed for 3 min and centrifuged at 4°C for 10 min at ×15,000 g to remove the solid particles in the supernatant. The supernatant was evaporated and dried through a centrifugal concentrator. The dried samples were re-dissolved in the initial mobile phase solutions, then vortex for 3 min and ultrasonic extraction for 5 min at 4°C. The samples were centrifuged twice at 4°C for 20 min at ×14,480 g, then the supernatant was transferred to analysis.

To validate the stability of the sequence analysis, a quality control (QC) sample was prepared by combining the same volume (10 μl) from each serum sample and then extracted and analyzed in the same way as the samples. The pooled QC sample and the blank (pure acetonitrile) sample were injected after every 10 samples during the analytical run to assess repeatability.

### 2.4 Magnetic Resonance Imaging Scans

After fixation, a 3% pentobarbital sodium solution (35 mg/kg) was injected intravenously into the ear margin of rabbits. After the anesthesia had taken effect, the bilateral femoral heads of all rabbits were scanned with a 3.0T magnetic resonance imaging (MRI) scanner (Achieva TX, Philips). The parameters were as follows: T_1_WI (TR 700 ms, TE 45 ms), T_2_WI (TR 2600 ms, TE 200 ms), Stir (TR 1500 ms, TE 60 ms), slice thickness 1.2 mm, flip angle of 90°, and matrix (reconstruction 256 mm × matrix scan 256 mm). Then the MRI results of SANFH in each group were compared.

### 2.5 Histopathology

Some rabbits were sacrificed for histopathological examination after collecting serum samples at the corresponding time points (1–6 weeks). After execution, the femoral head was removed aseptically and fixed with 10% formaldehyde solution and 0.1 mol/L phosphate buffer (pH 7.4) for 1 week. The bone specimens were then decalcified in 25% formic acid solution for 3 days, and then neutralized with 0.35 mol/L sodium sulfate for 3 days. The specimen was cut along the coronal plane and the distal (condyle) along the axial plane. The specimens were then embedded in paraffin, cut into thick sections of 5 mm, and stained with hematoxylin and eosin (H&E). Osteonecrosis of the femoral head was observed in H&E-stained samples under an optical microscope and microphotos were taken. Leica Co. The W550CW Signal Acquisition and Analysis System (Weztlar, Germany) was used for observation, analysis, and evaluation.

### 2.6 Ultra-High-Performance Liquid Chromatography Tandem Mass Spectrometry Analysis Conditions

UHPLC-MS/MS was used for the analysis of serum metabolites. The chromatographic column used was the Acquity™ UPLC BEH C18 column (2.1 mm × 100 mm, 1.7 µM) (Waters, United States). Both the positive and negative modes were selected as analytes. The UHPLC-MS/MS analysis uses the Q Exactive Plus High Resolution Mass Spectrometer (THERMO, United States) and was equipped with Ultimate 3,000 (DIONEX, TERMO, United States). The mass spectrometer operates in full mass spectrometry mode in the positive ionization mass range of 100–1500 M/Z. The chromatographic condition and the serum sample gradient were followed as previously described (Ren et al., 2018). The optimized chromatographic conditions were as follows: at the flow rate of 0.3 ml/min with a mobile phase consisting of isopropanol/acetonitrile 50:50 (V/V) with 0.1% formic acid (mobile phase A) and 0.1% formic acid solution (mobile phase B). Gradient elution process: 0–2 min, 2% A; 2–22 min, 2–99% A; 22–25 min, 99% A; 25.1–30 min, 2% A. The injection volume was 10 µl. Between two injections, the sampling needle was washed with 300 µl washing solution (methanol/water 50/50 V/V) once. The mass spectrometer was operated in full MS mode in the range of 100–1500 m/z positive ionization mass.

### 2.7 Data Processing and Statistical Analysis

UPLC-MS raw data was processed using an XCalibur Qual Browser (Thermo, United States). The raw data were transformed into Progensis QI (Waters, United States) for data analysis. The original data were pretreated with peak finding, alignment, filtering, and normalization. Principal component analysis (PCA) was applied to acquire an overview of the divergence in different groups. In multivariate analysis, rate analysis, partial least squares discriminant analysis (PLS-DA), and orthogonal partial least squares discriminant analysis (OPLS-DA) were performed using EZinfo 3.0 software. For the LC/MS dataset, the peaks were preliminarily identified by referring to online databases, such as alkaline lipids, glycerol lipids, lipid profiles, Metlin, and HMDB. The results obtained were consistent with the molecular ions and fragment ions in the experimental MS/MS spectra and were in agreement with those reported in the literature (Zhang et al., 2009; Zhu et al., 2011).

The HEML 1.0 software packages were applied to create a heat map to show the relative number of differential metabolites and to give their hierarchical cluster analysis (Deng et al., 2014; Ren et al., 2018). MetbraAnalyst 4.0 was used to analyze the pathways of the datasets of identified key metabolites. Results were presented as mean ± standard deviation (SD). Statistical significance was analyzed using Student’s t test, one-way analysis of variance (ANOVA), followed by Student-Newman-Keuls post hoc analysis. A *p-*value < 0.05 was considered a statistically significant difference.

## 3 Results

**FIGURE 1:**
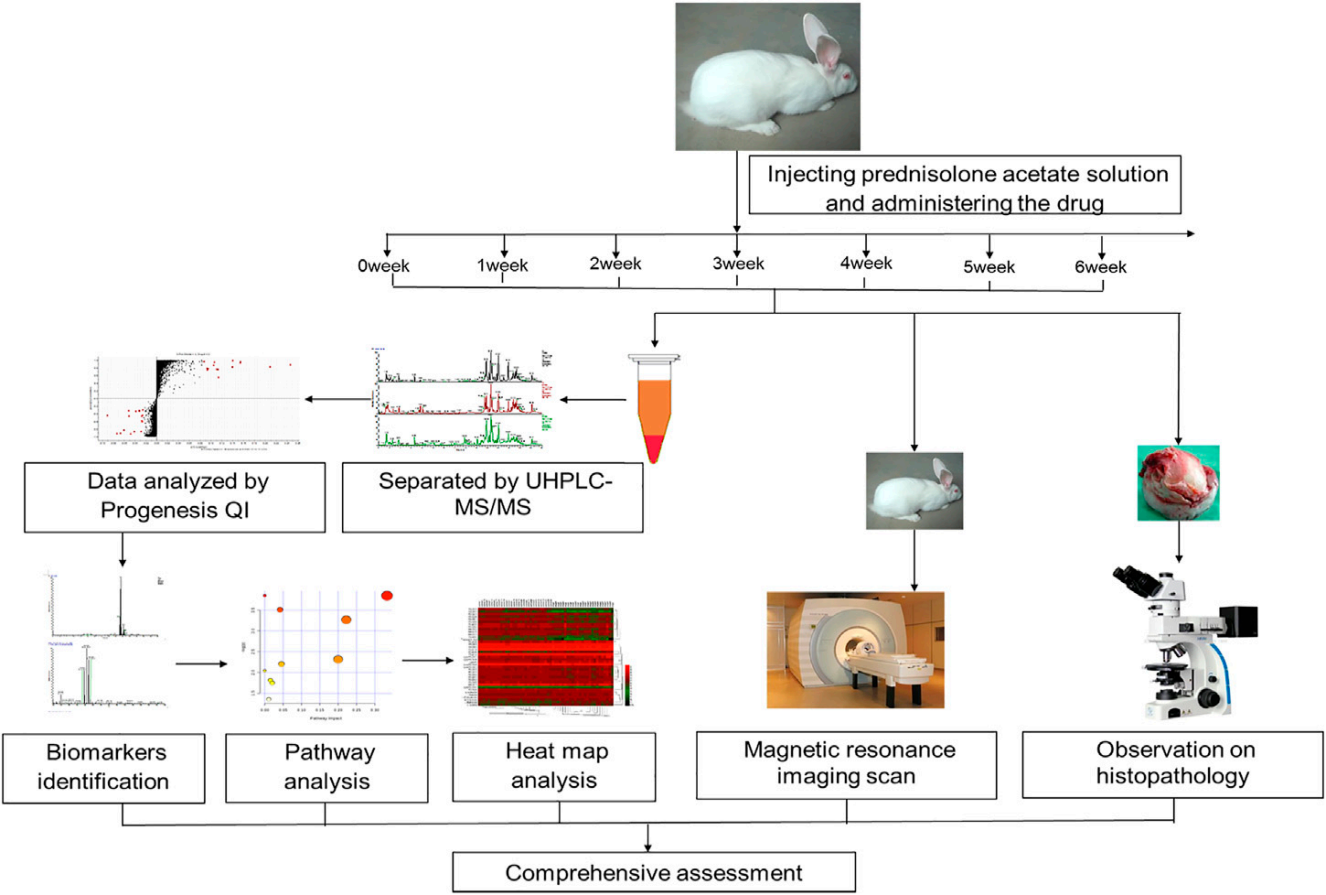
A schematic diagram of the discovery, identification, and pathways analysis of serum biomarkers in rabbits using un-targeted metabolomics based on UPLC-MS/MS, and the histopathology and imaging examination.

Figure 1 shows the general workflow of metabolite discovery, identification, pathways analysis, and GSLC efficacy determination in this study using the metabolomics strategy.

### 3.1 Gushiling Capsule Exerted Protective Effects on Glucocorticoid-Induced Osteonecrosis of the Femoral Head Rabbits

#### 3.1.1 Histopathologic Analysis

**FIGURE 2:**
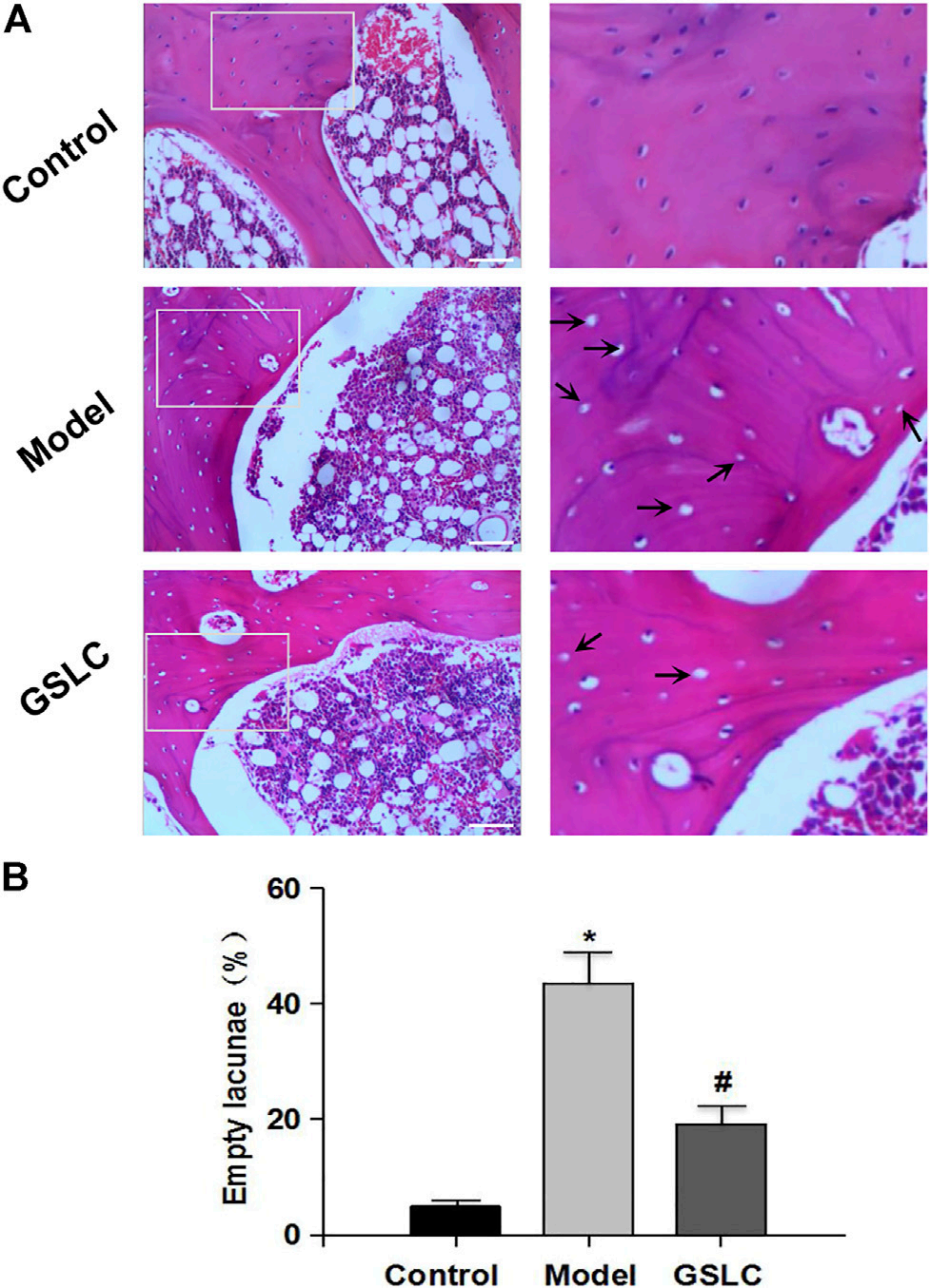
Histopathological analysis of GIONFH in vivo. The representative images of H&E staining from the femoral heads in three groups **(A)**. There were no empty lacunae in the femoral head of the control group. A large number of empty bone lacunae and necrotic bone marrow cells are visible in the model group, while there were few empty bone lacunae in the GSLC group. Scale bar = 100 μM. The histogram shows the proportion of empty bone lacunae in the model group, which are significantly higher than those in the control group and GSLC group **(B)**. The data are expressed as the means ± S.D. Significant differences between different groups are indicated as * *p* < 0.05 vs. the control group and # *p* < 0.05 vs. the model group.

Histopathological examination is an important standard for the diagnosis of GIONFH (Krenn et al., 2018; Crim et al., 2021). Based on the diagnostic criteria of GIONFH (Yamamoto et al., 1997; Dong et al., 2015; Yan et al., 2020), we evaluated femoral head specimens of rabbits using H&E staining. In this study, none of the rabbits in the control group developed osteonecrosis. Four of 25 rabbits in the GSLC group and 21 of 25 rabbits in the model group developed osteonecrosis; thus, the incidence of GIONFH was significantly decreased in the GSLC group compared with that in the model group. The percentage of empty lacunae in the model group was significantly higher than in the control group (Figure 2), and fractured trabeculae could be observed in the model group. Conversely, lower proportions of empty lacunae were observed in the GSLC group.

#### 3.1.2 Magnetic Resonance Imaging Results

**FIGURE 3:**
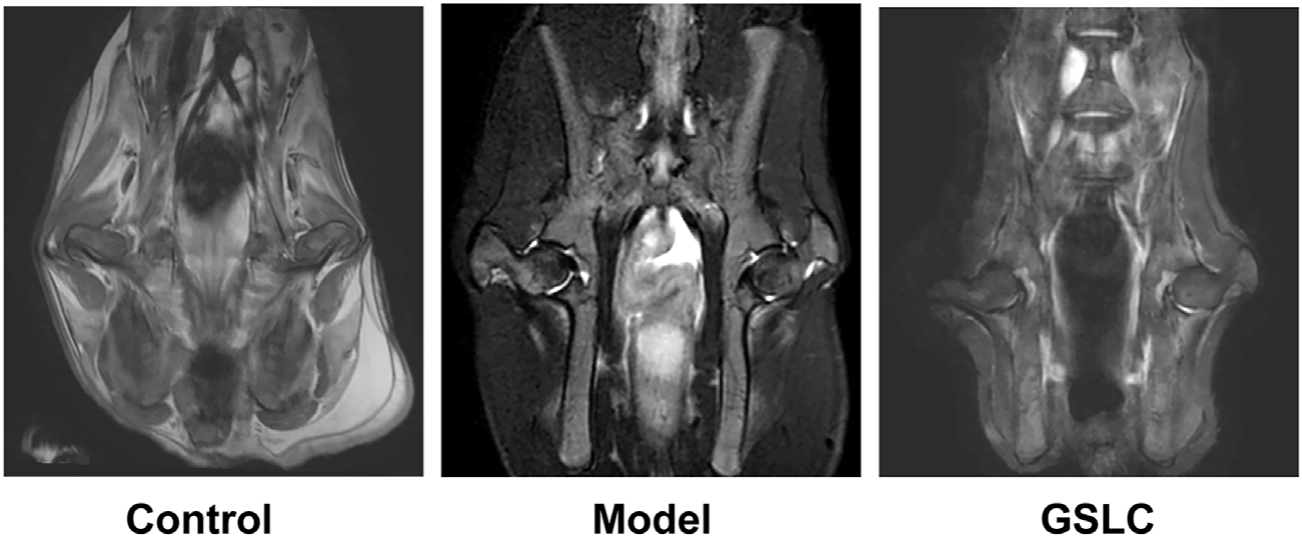
Analysis of bone structure of the rabbit femoral head by hip magnetic resonance coronal scanning. The representative images of rabbit bilateral femoral heads magnetic resonance imaging (MRI) scanning in control group, model group and GSLC group.

Magnetic resonance imaging (MRI) is considered the most sensitive, specific modality and gold standard for the diagnosis and evaluation of GIONFH (Chang et al., 2020; Zhang et al., 2021). MRI scanning was used to analyze the imaging changes in the rabbit femoral heads. In the control group, the MRI signals of the femoral head were normal and there was no abnormal signal in the soft tissue of the hip joint (Figure 3). In the model group, the T2-weighted images showed spotty, thin-thread, heterogeneous high-signal intensity, and the bilateral femoral heads were slightly collapsed and flattened (the right joint cavity, the bilateral hip joint space was not uniform). The GSLC group did not exhibit any significant abnormal MRI signals or obvious edema in the articular cavity (Figure 3). Collectively, these results indicated that GSLC could exert a protective effect on GIONFH *in vivo*.

### 3.2 Multivariate Analysis

**FIGURE 4:**
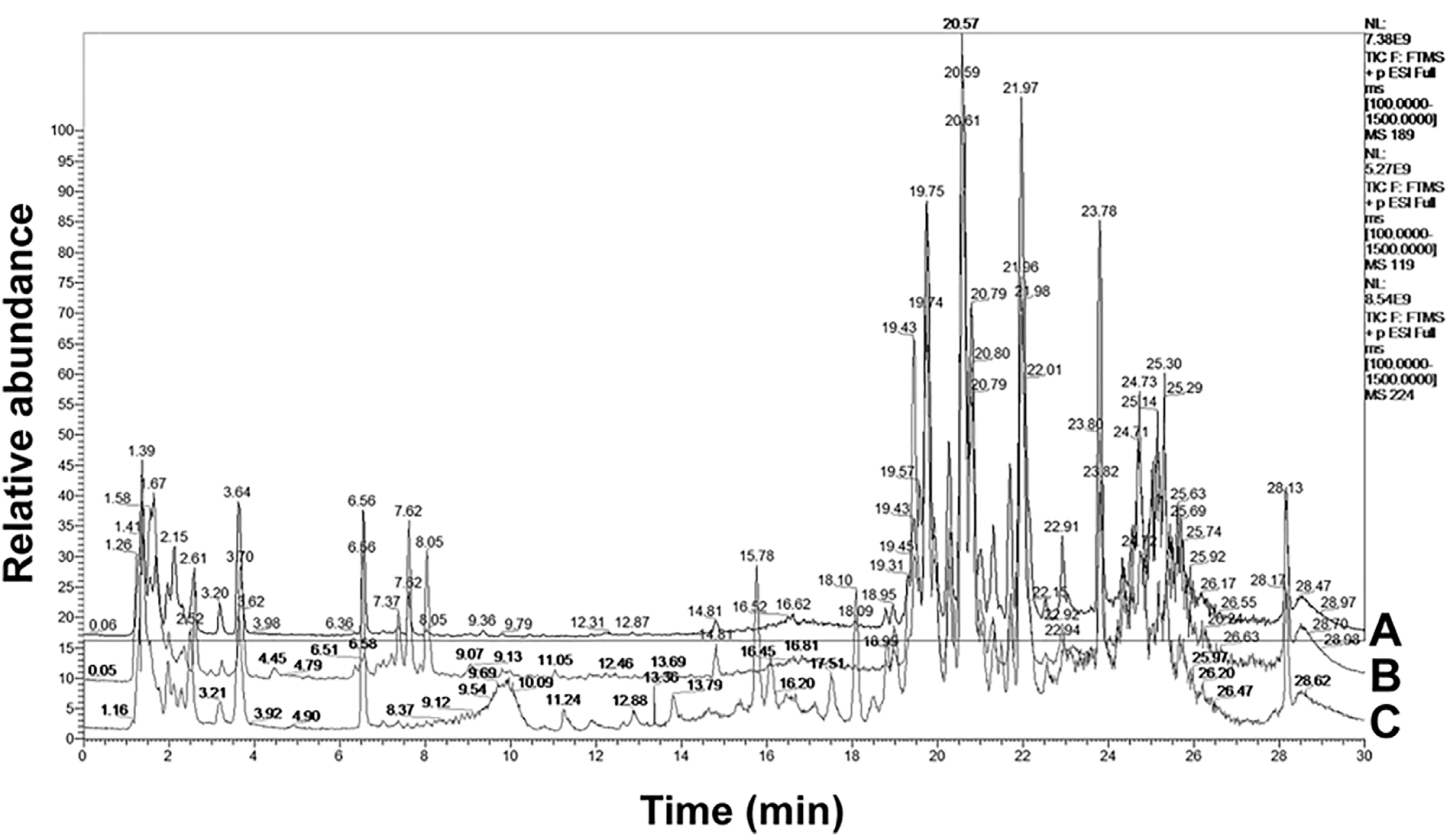
A typical LC-MS total ion current (TIC) chromatogram of rabbit serum samples from **(A)** the control group, **(B)** the model group and **(C)** the GSLC group.

A pooled quality control (QC) sample was repeatedly analyzed to evaluate the robustness and repeatability of the global serum metabolic spectrum analysis method based on LC-MS. We overlapped the total ion current (TIC) chromatograms of the QC sample, as shown in the Supplementary Figure S1, which demonstrated the high repeatability and the stability of our LC-MS system. The typical total ion current chromatograms of the serum metabolic profiles of the control group, model group, and GSLC group were analyzed by LC-MS shown in Figure 4. However, no significant differences were observed.

**FIGURE 5:**
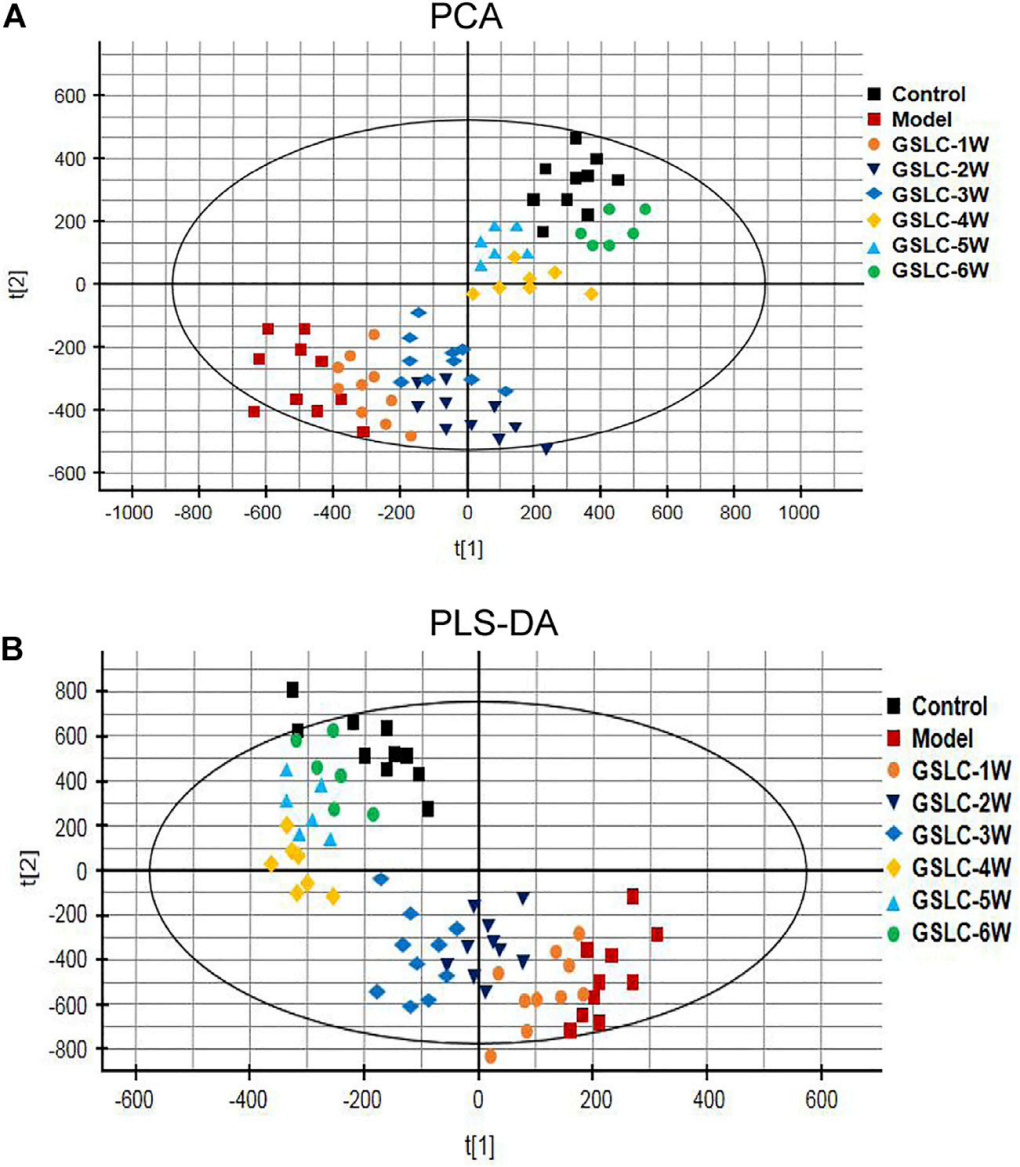
Multivariate statistical analysis of metabolic profiles derived from the control group, model group and GSLC groups (1–6 weeks). **(A)** Principal component analysis (PCA) score plots of the serum samples from control group, model group and GSLC groups. **(B)** Partial least squares-discriminant analysis (PLS-DA) score plots of the serum samples from control, model, and GSLC groups.

Subtle changes could be found using a pattern recognition approach, such as principal component analysis (PCA) and partial least-squares discriminate analysis (PLS-DA). Therefore, PCA was used to perform unsupervised data analysis on the control, model, and GSLC groups. Most of these groups could be easily distinguished from each other. As shown in Figure 5A, there was a net separation between the control group, model group and GSLC groups (1–6 weeks) in the PCA score plot. The results reflected the successful modeling process and GSLC caused obvious endogenous metabolite changes after GIONFH. Partial least squares-discriminate analysis (PLS-DA) and orthogonal PLS-DA (OPLS-DA) were used to verify the model and to explore the different metabolites between groups. PLS-DA is more appropriate than PCA for classification, and OPLS-DA is most effective in separating two groups (Jones et al., 2014). Therefore, to discriminate differentially expressed metabolites between each pair of groups, a supervised PLS-DA analysis was performed to classify the control group, model group, and GSLC groups (1–6 weeks). The trend of different time points after GSLC administration provided intuitive findings used to evaluate the therapeutic effect. When rabbits were administered GSLC for 1, 2, or 3 weeks, the serum sample spots of rabbits were similar, despite being separated. However, when rabbits were treated with GSLC for 4–6 weeks, endogenous metabolites changed and were significantly separated from the model group, but had a tendency to return to a profile more similar to that of the control group (Figure 5B). The quality of the model was described using the goodness-of-fit parameter R^2^Y, and the predictive ability parameter Q^2^. The cumulative R^2^Y and Q^2^ for PLS-DA model were 0.95 and 0.84, respectively, which demonstrated robustness and good predictive ability of the model.

**FIGURE 6:**
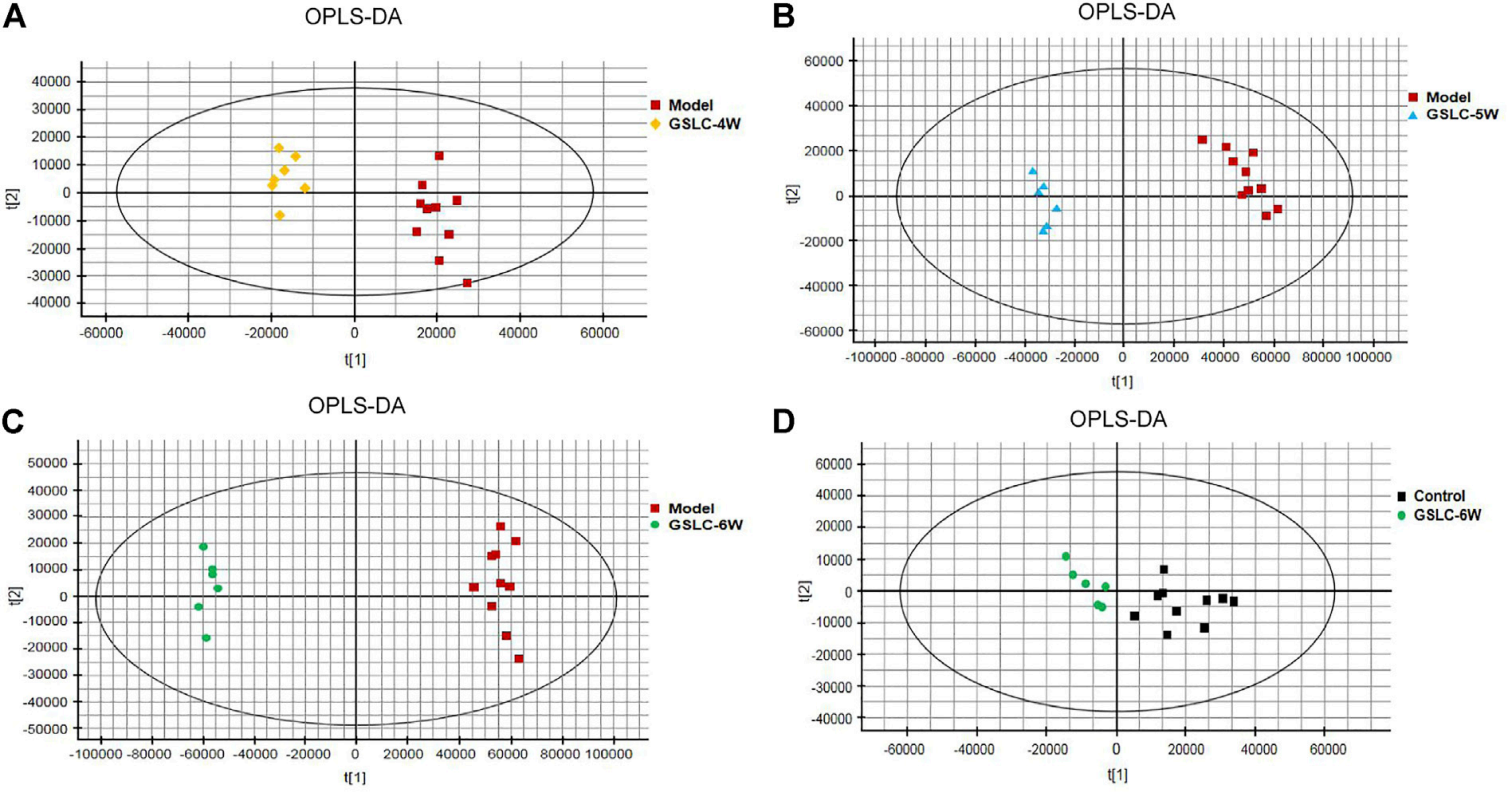
Multivariate statistical analysis of metabolic profiles derived from the control, model, and GSLC group. **(A-D)** Orthogonal partial least squares-discriminant analysis (OPLS-DA) score plots of the serum samples from control group, model group and GSLC groups (4–6 weeks).

Although the PCA and PLS-DA score plots provided an overview of these groups, the variables responsible for the differences in each group were still unclear. To further determine whether GSLC influenced the metabolic pattern of GIONFH rabbits, a supervised OPLS-DA model was built to identify potential markers for the pathological process of GIONFH. The OPLS-DA analysis was responsible for the intergroup separation. As shown in Figures 6A–C, the metabolomic profiles of the GSLC treatment groups at 4–6 weeks were distinctly separated from those of the model group. However, the metabolomic profiles of the GSLC administration for 6 weeks showed a tendency to be similar to the control group (Figure 6D). These results indicated that GSLC exerted a positive effect on metabolism in GIONFH rabbits. To estimate the predictive ability of our model, the cross-validation was used to validate the OPLS-DA models. In theory, the values of R^2^Y and Q^2^ should be close to 1, which represents good stability and high predictability (Gromski et al., 2015). The S-plots of serum samples analysis in different phases and the Goodness-of-Fit are shown in Supplementary Figure S2. In the OPLS-DA model, the values of R^2^Y and Q^2^ were close to 1.0, suggesting that the models established in this study had good fitness and prediction.

### 3.3 Identification of Potential Biomarkers

Biomarkers play an important role in the diagnosis and determination of the prognosis of diseases. Based on the tag of the S plots and *p* < 0.05, CV < 0.3, the representative metabolites were detected between the GSLC group and the model group. In this study, biomarkers were identified on the basis of accurate mass measurements via UHPLC-MS/MS and data comparison was performed with theoretical isotopic patterns and databases, such as the Human Metabolome Database, Metlin Database, LIPID MAPS, and ChemSpider database (Ren et al., 2018). To simplify the process of identifying potential biomarkers, we selected the ion at *t*_*R*_ = 19.26 min (*m/z* 494.3235) as an example that will be described in Supplementary Figure S3. Furthermore, to analyze the overall differential expression of potential biomarkers, a heat map was constructed using the Heml (Version 1.0) software package according to a ratio of the content of each biomarker in each group to directly compare the relationship among the different groups (Supplementary Figure S4).

### 3.4 Gushiling Capsule Regulated Metabolic Profiles in Glucocorticoid-Induced Osteonecrosis of the Femoral Head Rabbits

**FIGURE 7:**
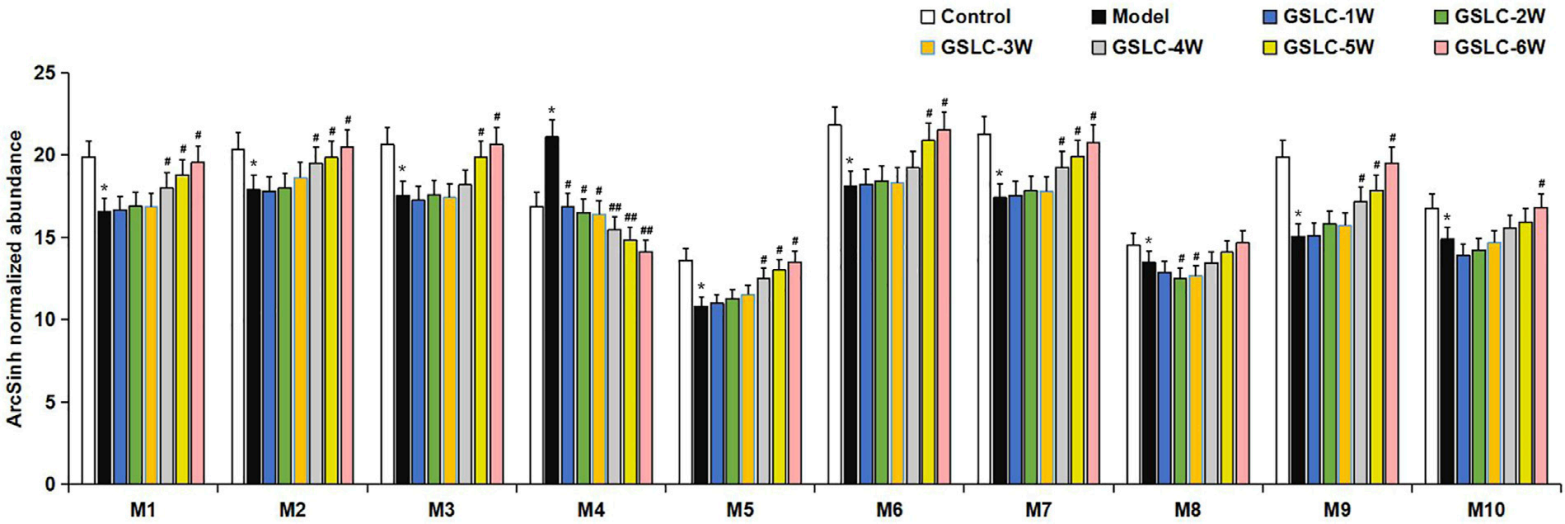
The changes of relative intensity of target potential metabolites identified in serum samples in different groups. Significant differences between different groups are indicated as * *p* < 0.05 vs. the control group and # *p* < 0.05, ## *p* < 0.01 vs. the model group. M1, PC (40:6); M2, PC (38:7); M3, LysoPC (14:0); M4, LysoPC (20:4); M5, valerylcarnitine; M6, LysoPC (16:1); M7, LysoPC (18:1); M8, tetracosahexaenoic acid; M9, LysoPC (22:6); M10, PC (40:8).

According to the protocol above, ten endogenous metabolites in the serum were tentatively identified. The changes in potential biomarkers levels across different groups are shown in Figure 7. The normalized abundance of each metabolite was transformed using the arcsinh function because the distributions were skewed. The arcsinh is an incremental function and describes the trend of contents of metabolites in each group. Compared to the control group, the content of PC (40:6), LysoPC (14:0), LysoPC (16:1), LysoPC (22:6), PC (38:7), LysoPC (18:1), PC (40:8), tetracosahexaenoic acid, and valerylcarnitine decreased significantly in the model groups. LysoPC (20:4) increased significantly in GIONFH model rabbits compared to the control group. After administration with GSLC, the above potential markers showed different levels of recovery compared to that in the control group, especially GSLC treatment for 4–6 weeks. These results suggested that GSLC could regulate metabolic profiles, mainly of phospholipids, and could improve disordered phospholipid metabolism in GIONFH rabbits.

### 3.5 Pathway Analysis

**FIGURE 8:**
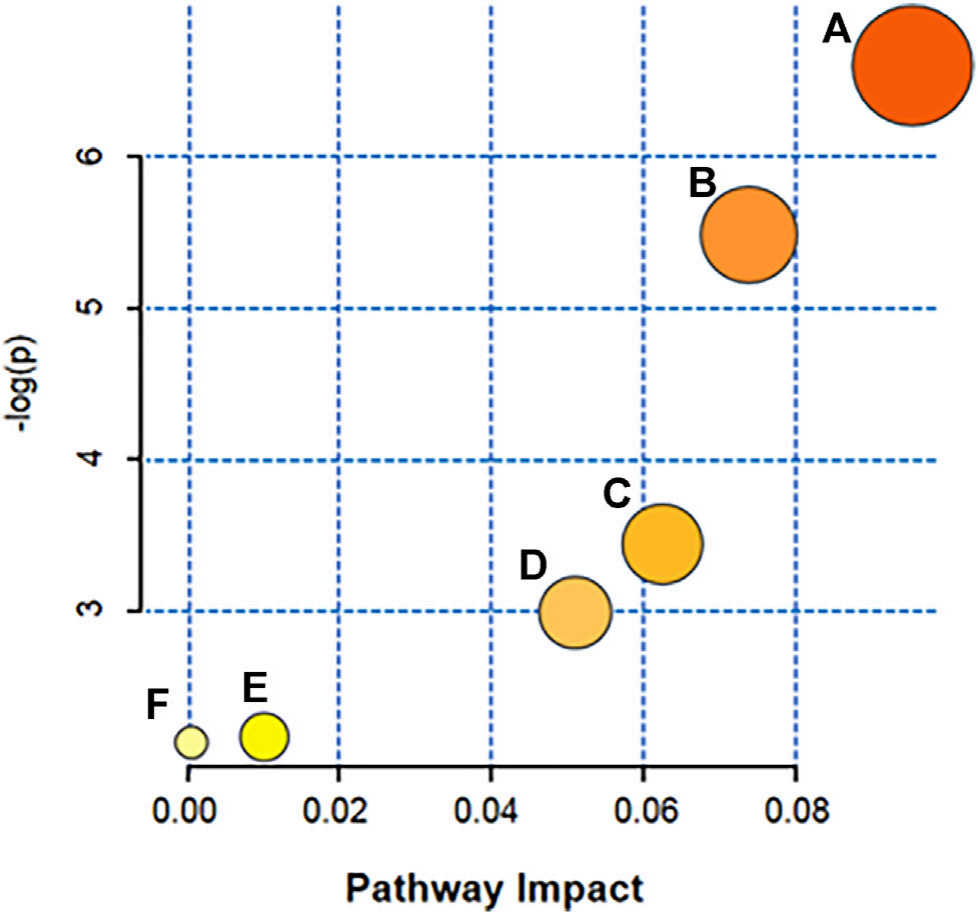
Metabolic pathway analysis for serum samples in the GIONFH rabbit model using MetaboAnalyst4.0. The size and colored shades of the dots were positively associated with the effects on metabolic pathways, and each spot represented a metabolic pathway. **(A)** glycerophospholipid metabolism, **(B)** sphingolipid metabolism, **(C)** linoleic acid metabolism, **(D)** alpha-Linolenic acid metabolism, **(E)** pyrimidine metabolism, **(F)** arachidonic acid metabolism.

To reveal metabolite pathways and their metabolic processes after GSLC administration, the identified serum metabolites were introduced into MetaboAnalyst4.0 (https://www.metaboanalyst.ca/) for pathway analysis. As shown in Figure 8, GIONFH was associated with six metabolic pathways, including glycerophospholipid metabolism, sphingolipid metabolism, linoleic acid metabolism, alpha-linolenic acid metabolism, pyrimidine metabolism, and arachidonic acid metabolism. The details of the pathways are listed in Table 1. These pathways with an impact value > 0.05 were considered the most relevant pathways involved in GIONFH. As a result, four main metabolic pathways were selected: glycerophospholipid metabolism, sphingolipid metabolism, linoleic acid metabolism and alpha-linolenic acid metabolism. These pathways are suggested to be potential pathways for the targeted intervention of GSLC against GIONFH.

**TABLE 1:** Metabolic pathway analysis performed using MetbraAnalyst 4.0 revealing six pathways.

No	Metabolic pathways	Impact	*p-*value	-log(*p*)
1	glycerophospholipid metabolism	0.0965	0.0153	6.7025
2	sphingolipid metabolism	0.0778	0.0391	5.3995
3	linoleic acid metabolism	0.0626	0.0427	3.3905
4	alpha-Linolenic acid metabolism	0.0510	0.0487	3.0174
5	pyrimidine metabolism	0.0127	0.4631	0.7448
6	arachidonic acid metabolism	0.0013	0.5138	0.6534

## 4 Discussion

GIONFH is a widespread and intractable disease with a high disability rate, which necessitates novel therapeutic options. In clinical practice, GSLC is an effective traditional Chinese herbal formula for the treatment of GIONFH. The purpose of this study was to determine the therapeutic effects of GSLC on GIONFH, and to clarify the related mechanisms using a nontargeted metabolomics approach.

With the widespread clinical application of dexamethasone and other glucocorticoids, GIONFH has become a commonly occurring type of non-traumatic femoral head necrosis (Xu et al., 2014). Although the pathogenesis of GIONFH remains uncertain, several factors such as lipid metabolism disorder, cellular apoptosis, intravascular coagulation, microvascular injury, osteoporosis, and intraosseous hypertension have been postulated to play a role in GIONFH (Kubo et al., 2016; Zhang et al., 2020; Xu et al., 2021). Abnormal lipid metabolism is an important factor that leads to induction of ONFH by GC treatment through the activation of lipid peroxidation (Iio et al., 1996). Long-term and excessive use of hormones causes changes in the microenvironment of the femoral head, resulting in the overexpression of factors related to apoptosis such as peroxide lipid, Bax, caspase-3, and caspase-9 (Islam et al., 1997; Qiang et al., 2015). These apoptotic factors cause cell apoptosis and tissue destruction through a series of reactions. In this study, we showed that the percentage of empty lacunae in the model group was significantly higher than that in the control group, but lower proportions of empty lacunae were observed in the GSLC group. Furthermore, MRI findings showed that the GSLC group did not exhibit any significantly abnormal signals or non-obvious edema in the articular cavity. Taken together, these results indicated that GSLC could play a protective effect on GIONFH *in vivo*.

GSLC, a Chinese medicinal formula, has been used clinically to treat GIONFH for many years. There have been some reports on the regulation of lipid metabolism of the major components of GSLC. For instance, *Salvia miltiorrhiza Bunge* [Lamiaceae] and *Conioselinum anthriscoides* “*Chuanxiong*” [Apiaceae] could regulate lipid metabolism and improve abnormal lipid metabolism (Dong et al., 2018; Zhang et al., 2019; Shen et al., 2021). This study showed that GSLC could regulate metabolic profiles, mainly phospholipids, to improve the metabolism of disordered phospholipids. Furthermore, *in vivo* experiments showed that GSLC could reduce the incidence of GIONFH in rabbits.

Metabolomics is an emerging and powerful discipline that involves the comprehensive analysis of small molecules and provides a powerful method to discover biomarkers in biological systems (Wang et al., 2012). Metabolomics can be divided into un-targeted and targeted metabolomics (Cui et al., 2018). Un-targeted metabolomics has the advantages of high efficiency, sensitivity, and comprehensiveness, which has led it to be increasingly used to reveal the complex action mechanism of traditional Chinese medicine (Guijas et al., 2018). Different analytical methods that involve multivariate data analysis, such as PCA, PLS-DA, and OPLS-DA, have been applied in metabolomic-based drug metabolism studies (Zhang et al., 2012). In this study, non-targeted UHPLC-MS/MS-based metabolomics was used to identify key biomarkers, metabolic pathways and mechanisms of action of GSLC in the prevention and treatment of GIONFH in rabbits. The results of the multivariate statistical analysis indicated that GSLC had a beneficial effect on metabolism in GIONFH rabbits.

Phospholipids play a central role in the biochemistry of all living organisms, constituting the lipid bilayer that serves as a structural barrier to protect cells and subcellular components from external conditions and are required for the proper function of integral membrane proteins, receptors, and ion channels (Spector and Yorek, 1985). Phospholipid metabolism is an essential regulator of apoptosis. Lipoprotein mediators, produced from phospholipids, have been reported to be critical influencers of apoptosis (Wójcik et al., 2020). Farooqui et al. reported that lysophospholipids are intermediates in phospholipid metabolism and are involved in cell membrane lysis, apoptosis, and inflammatory responses (Farooqui et al., 2000). Increasing evidence has shown that GIONFH is associated with a disorder of lipid or fatty acid metabolism (Miyanishi et al., 1999; Okazaki et al., 2009; Wang et al., 2021). However, changes and effects of phospholipid metabolism on GIONFH remain unknown. In the present study, the UHPLC-MS/MS-based serum metabolomics method combined with a multivariate statistical analysis results identified lower levels of 10 metabolites, namely LysoPC (14:0), LysoPC (16:1), LysoPC (22:6), PC (38:7), PC (36:1), LysoPC (18:1), PC (40:8), LysoPC (20:4), tetracosahexaenoic acid, and valeryl carnitine, which were determined to be potential biomarkers associated with GIONFH. Furthermore, analysis of the metabolic pathways of these metabolites suggested that glycerophospholipid metabolism, linoleic acid metabolism, sphingolipid metabolism, and alpha-linolenic acid metabolism were the metabolic pathways most significantly altered in GIONFH rabbits. Taken together, these results suggested that GSLC could regulate metabolic profiles, mainly those of phospholipids, and could re-adjusted these metabolically disordered phospholipid levels, and thus exert a therapeutic role in GIONFH.

Some limitations of the study should be noted. First, only one rabbit animal model was used for the *in vivo* experiments. Various animal species have been used as experimental models for GIONFH, including rats, rabbits, dogs, pigs, emu, and sheep (Xu et al., 2018). Furthermore, we investigated the metabolic profiles of serum samples from GIONFH rabbits to identify potential biomarkers and study the effect of GSLC on GIONFH rabbits, but there is a lack of sample data from clinical patients. In addition, we did not use classical molecular biological methods, such as PCR and Western blot, to demonstrate the mechanism of GSLC. Therefore, it may be difficult to illustrate the mechanism of GIONFH in humans, and further studies are needed.

In this study, histopathological analysis and MRI scan results revealed a remarkable therapeutic effect of GSLC against GIONFH in rabbits. Subsequently, 10 potential biomarkers and 6 metabolic pathways were identified during the GIONFH process using metabolomics based on UHPLC-MS/MS coupled with multivariate statistical analyses. Our findings indicated that GSLC could restore disordered metabolic profiles, mainly involving phospholipids, and thus exerting a therapeutic effect on GIONFH. We also found that these metabolites were mainly related to glycerophospholipid metabolism, sphingolipid metabolism, linoleic acid metabolism, and alpha-linolenic acid metabolism *in vivo*, which fully illustrated the overall effect of GSLC in the prevention and treatment of GIONFH through multiple pathways, multiple levels, and multiple targets. To our knowledge, this study is the first to use an un-targeted metabolomics method to clarify the drug effects of GSLC in GIONFH rabbits. Our study provides a reference for the pathogenesis of GIONFH and the preventive and therapeutic effect of GSLC and provides more support for its clinical application in traditional Chinese medicine.

## Data Availability

The original contributions presented in the study are included in the article/Supplementary Material, further inquiries can be directed to the corresponding author.

## References

[B1] Bai R. Feng W. Liu W. L. Zhao Z. H. Zhao A. Q. Wang Y. (2016). Roles of Osteocyte Apoptosis in Steroid-Induced Avascular Necrosis of the Femoral Head. Genet. Mol. Res. 15 (1), 15017529. 10.4238/gmr.15017529 27050956

[B2] Chang C. Greenspan A. Gershwin M. E. (2020). The Pathogenesis, Diagnosis and Clinical Manifestations of Steroid-Induced Osteonecrosis. J. Autoimmun. 110, 102460. 10.1016/j.jaut.2020.102460 32307211

[B3] Chaudhry F. Kawai H. Johnson K. W. Narula N. Shekhar A. Chaudhry F. (2020). Molecular Imaging of Apoptosis in Atherosclerosis by Targeting Cell Membrane Phospholipid Asymmetry. J. Am. Coll. Cardiol. 76 (16), 1862–1874. 10.1016/j.jacc.2020.08.047 33059832 PMC7654709

[B4] Chen C. Shah Y. M. Morimura K. Krausz K. W. Miyazaki M. Richardson T. A. (2008). Metabolomics Reveals that Hepatic Stearoyl-CoA Desaturase 1 Downregulation Exacerbates Inflammation and Acute Colitis. Cell Metab 7 (2), 135–147. 10.1016/j.cmet.2007.12.003 18249173 PMC2276699

[B5] Chen C. Y. Du W. Rao S. S. Tan Y. J. Hu X. K. Luo M. J. (2020). Extracellular Vesicles from Human Urine-Derived Stem Cells Inhibit Glucocorticoid-Induced Osteonecrosis of the Femoral Head by Transporting and Releasing Pro-angiogenic DMBT1 and Anti-apoptotic TIMP1. Acta Biomater. 111, 208–220. 10.1016/j.actbio.2020.05.020 32447063

[B6] Chen F. Hao L. Zhu S. Yang X. Shi W. Zheng K. (2021). Potential Adverse Effects of Dexamethasone Therapy on COVID-19 Patients: Review and Recommendations. Infect. Dis. Ther. 10 (4), 1907–1931. 10.1007/s40121-021-00500-z 34296386 PMC8298044

[B7] Cheng B. Zheng H. Wu F. Wu J. Liu X. Tang C. (2017). Metabolomics Analysis of Danggui Sini Decoction on Treatment of Collagen-Induced Arthritis in Rats. J. Chromatogr. B Analyt Technol. Biomed. Life Sci. 1061-1062, 282–291. 10.1016/j.jchromb.2017.07.043 28763759

[B8] Crim J. Layfield L. J. Stensby J. D. Schmidt R. L. (2021). Comparison of Radiographic and Pathologic Diagnosis of Osteonecrosis of the Femoral Head. AJR Am. J. Roentgenol 216 (4), 1014–1021. 10.2214/ajr.20.22930 33534621

[B9] Cui L. Lu H. Lee Y. H. (2018). Challenges and Emergent Solutions for LC-MS/MS Based Untargeted Metabolomics in Diseases. Mass. Spectrom. Rev. 37 (6), 772–792. 10.1002/mas.21562 29486047

[B10] Deng W. Wang Y. Liu Z. Cheng H. Xue Y. (2014). HemI: a Toolkit for Illustrating Heatmaps. PloS one 9 (11), e111988. 10.1371/journal.pone.0111988 25372567 PMC4221433

[B11] Dong X. L. Yu W. X. Li C. M. He S. Zhou L. P. Poon C. W. (2018). Danshen (Salvia Miltiorrhiza) Protects Ovariectomized Rats Fed with High-Saturated Fat-Sucrose Diet from Bone Loss. Osteoporos. Int. 29 (1), 223–235. 10.1007/s00198-017-4254-2 29058051

[B12] Dong Y. Li Y. Huang C. Gao K. Weng X. (2015). Systemic Application of Teriparatide for Steroid Induced Osteonecrosis in a Rat Model. BMC Musculoskelet. Disord. 16, 163. 10.1186/s12891-015-0589-z 26163144 PMC4499203

[B13] Fan W. Yu X. Ren X. Chen K. (2018). Intervention Research on Chinese Medicine Gushiling Capsule in Treating Steroid-Associated Necrosis of the Femoral Head in Rabbits. J. Pract. Med. 34 (13), 5. 10.3969/j.issn.1006-5725.2018.13.012

[B14] Farooqui A. A. Horrocks L. A. Farooqui T. (2000). Glycerophospholipids in Brain: Their Metabolism, Incorporation into Membranes, Functions, and Involvement in Neurological Disorders. Chem. Phys. Lipids 106 (1), 1–29. 10.1016/s0009-3084(00)00128-6 10878232

[B15] Gromski P. S. Muhamadali H. Ellis D. I. Xu Y. Correa E. Turner M. L. (2015). A Tutorial Review: Metabolomics and Partial Least Squares-Discriminant Analysis-Aa Marriage of Convenience or a Shotgun Wedding. Anal. Chim. Acta 879, 10–23. 10.1016/j.aca.2015.02.012 26002472

[B16] Guijas C. Montenegro-Burke J. R. Warth B. Spilker M. E. Siuzdak G. (2018). Metabolomics Activity Screening for Identifying Metabolites that Modulate Phenotype. Nat. Biotechnol. 36 (4), 316–320. 10.1038/nbt.4101 29621222 PMC5937131

[B17] Guo X. D. Liu L. Xiao H. Y. (2018). High-throughput Metabolomics for Discovering Metabolic Biomarkers from Intestinal Tumorigenesis in APC Min/+ Mice Based on Liquid Chromatography/mass Spectrometry. J. Chromatogr. B Analyt Technol. Biomed. Life Sci. 1100-1101, 131–139. 10.1016/j.jchromb.2018.09.042 30316137

[B18] Hongtao L. I. Cheng Y. Sun X. Wang B. Wang S. (2015). Effect of Gushiling Capsule on VEGFmRNA Expression in Bone of Early Steroid- Induced Necrosis of Femoral Head in Rabbits. Chin. J. Traditional Med. Sci. Tech. 22 (04), 390–397.

[B19] Hu J. Lipowsky R. Weikl T. R. (2013). Binding Constants of Membrane-Anchored Receptors and Ligands Depend Strongly on the Nanoscale Roughness of Membranes. Proc. Natl. Acad. Sci. U S A. 110 (38), 15283–15288. 10.1073/pnas.1305766110 24006364 PMC3780905

[B20] Huang M. Wang P. Xu S. Xu W. Xu W. Chu K. (2015). Biological Activities of Salvianolic Acid B from Salvia Miltiorrhiza on Type 2 Diabetes Induced by High-Fat Diet and Streptozotocin. Pharm. Biol. 53 (7), 1058–1065. 10.3109/13880209.2014.959611 25612777

[B21] Iio H. Ake Y. Saegusa Y. Mizuno K. (1996). The Effect of Lipid Peroxide on Osteoblasts and Vascular Endothelial Cells-Tthe Possible Role of Ischemia-Reperfusion in the Progression of Avascular Necrosis of the Femoral Head. Kobe J. Med. Sci. 42 (6), 361–373. 9153973

[B22] Islam D. Veress B. Bardhan P. K. Lindberg A. A. Christensson B. (1997). *In Situ* characterization of Inflammatory Responses in the Rectal Mucosae of Patients with Shigellosis. Infect. Immun. 65 (2), 739–749. 10.1128/iai.65.2.739-749.1997 9009337 PMC176120

[B23] Jiang Y. Zhang Y. Zhang H. Zhu B. Li P. Lu C. (2014). Pravastatin Prevents Steroid-Induced Osteonecrosis in Rats by Suppressing PPARγ Expression and Activating Wnt Signaling Pathway. Exp. Biol. Med. (Maywood) 239 (3), 347–355. 10.1177/1535370213519215 24510055

[B24] Jiang Z. Liang Q. Luo G. Hu P. Li P. Wang Y. (2009). HPLC-electrospray Tandem Mass Spectrometry for Simultaneous Quantitation of Eight Plasma Aminothiols: Application to Studies of Diabetic Nephropathy. Talanta 77 (4), 1279–1284. 10.1016/j.talanta.2008.08.031 19084635

[B25] Jin S. Meng C. He Y. Wang X. Zhang Q. Wang Z. (2020). Curcumin Prevents Osteocyte Apoptosis by Inhibiting M1-type Macrophage Polarization in Mice Model of Glucocorticoid-Associated Osteonecrosis of the Femoral Head. J. Orthop. Res. 38 (9), 2020–2030. 10.1002/jor.24619 32009245 PMC7496963

[B26] Jing X. Du T. Yang X. Zhang W. Wang G. Liu X. (2020). Desferoxamine Protects against Glucocorticoid-Induced Osteonecrosis of the Femoral Head via Activating HIF-1α Expression. J. Cel Physiol 235 (12), 9864–9875. 10.1002/jcp.29799 32437020

[B27] Jones A. E. Turner P. Zimmerman C. Goulermas J. Y. (2014). Classification of Spent Reactor Fuel for Nuclear Forensics. Anal. Chem. 86 (11), 5399–5405. 10.1021/ac5004757 24805973

[B28] Krenn V. Müller S. Krenn V. T. Hempfling H. (2018). Pathophysiology of Aseptic Femoral Head Necrosis: Pathogenesis and Histopathological Differential Diagnosis. Orthopade 47 (9), 710–716. 10.1007/s00132-018-3608-6 30062451

[B29] Kubo T. Ueshima K. Saito M. Ishida M. Arai Y. Fujiwara H. (2016). Clinical and Basic Research on Steroid-Induced Osteonecrosis of the Femoral Head in Japan. J. Orthop. Sci. 21 (4), 407–413. 10.1016/j.jos.2016.03.008 27062553

[B30] Ledford H. (2020). How Does COVID-19 Kill? Uncertainty Is Hampering Doctors' Ability to Choose Treatments. Nature 580 (7803), 311–312. 10.1038/d41586-020-01056-7 32273618

[B31] Leslie R. D. Beyan H. (2011). Metabolomics Makes a Mark: Early Changes Associated with Autoimmune Diabetes. Diabetes 60 (11), 2688–2690. 10.2337/db11-1177 22025776 PMC3198102

[B32] Li Y. M. Wang S. X. Gao H. S. Wang J. G. Wei C. S. Chen L. M. (2004). Factors of Avascular Necrosis of Femoral Head and Osteoporosis in SARS Patients' Convalescence. Zhonghua Yi Xue Za Zhi 84 (16), 1348–1353. 15387943

[B33] Miyanishi K. Yamamoto T. Irisa T. Noguchi Y. Sugioka Y. Iwamoto Y. (1999). Increased Level of Apolipoprotein B/apolipoprotein A1 Ratio as a Potential Risk for Osteonecrosis. Ann. Rheum. Dis. 58 (8), 514–516. 10.1136/ard.58.8.514 10419872 PMC1752928

[B34] Newgard C. B. An J. Bain J. R. Muehlbauer M. J. Stevens R. D. Lien L. F. (2009). A Branched-Chain Amino Acid-Related Metabolic Signature that Differentiates Obese and Lean Humans and Contributes to Insulin Resistance. Cel Metab 9 (4), 311–326. 10.1016/j.cmet.2009.02.002 PMC3640280 19356713

[B35] Okazaki S. Nishitani Y. Nagoya S. Kaya M. Yamashita T. Matsumoto H. (2009). Femoral Head Osteonecrosis Can Be Caused by Disruption of the Systemic Immune Response via the Toll-like Receptor 4 Signalling Pathway. Rheumatology (Oxford) 48 (3), 227–232. 10.1093/rheumatology/ken462 19129349

[B36] Patterson A. D. Maurhofer O. Beyoglu D. Lanz C. Krausz K. W. Pabst T. (2011). Aberrant Lipid Metabolism in Hepatocellular Carcinoma Revealed by Plasma Metabolomics and Lipid Profiling. Cancer Res. 71 (21), 6590–6600. 10.1158/0008-5472.can-11-0885 21900402 PMC3206149

[B37] Qiang H. Liu H. Ling M. Wang K. Zhang C. (2015). Early Steroid-Induced Osteonecrosis of Rabbit Femoral Head and Panax Notoginseng Saponins: Mechanism and Protective Effects. Evid. Based Complement. Alternat Med. 2015, 719370. 10.1155/2015/719370 25866538 PMC4378605

[B38] Ren X. Fan W. Shao Z. Chen K. Yu X. Liang Q. (2018). A Metabolomic Study on Early Detection of Steroid-Induced Avascular Necrosis of the Femoral Head. Oncotarget 9 (8), 7984–7995. 10.18632/oncotarget.24110.18632/oncotarget.24150 29487708 PMC5814275

[B39] Rhee E. P. Gerszten R. E. (2012). Metabolomics and Cardiovascular Biomarker Discovery. Clin. Chem. 58 (1), 139–147. 10.1373/clinchem.2011.169573 22110018 PMC4402975

[B40] Segawa K. Kurata S. Yanagihashi Y. Brummelkamp T. R. Matsuda F. Nagata S. (2014). Caspase-mediated Cleavage of Phospholipid Flippase for Apoptotic Phosphatidylserine Exposure. Science 344 (6188), 1164–1168. 10.1126/science.1252809 24904167

[B41] Shen J. Liang B. L. Zeng Q. S. Chen J. Y. Liu Q. Y. Chen R. C. (2006). Investigation of Proximal Femoral Marrow with Magnetic Resonance Imaging in Recovered Patients with Severe Acute Respiratory Syndrome. Zhonghua Jie He He Hu Xi Za Zhi 29 (3), 189–193. 16677484

[B42] Shen W. Jin B. Han Y. Wang H. Jiang H. Zhu L. (2021). The Effects of Salvia Miltiorrhiza on Reproduction and Metabolism in Women with Polycystic Ovary Syndrome: A Systematic Review and Meta-Analysis. Evid. Based Complement. Alternat Med. 2021, 9971403. 10.1155/2021/9971403 34055030 PMC8143891

[B43] Song Y. Du Z. Chen B. Ren M. Yang Q. Sui Y. (2017). Association of SREBP2 Gene Polymorphisms with the Risk of Osteonecrosis of the Femoral Head Relates to Gene Expression and Lipid Metabolism Disorders. Mol. Med. Rep. 16 (5), 7145–7153. 10.3892/mmr.2017.7473 28901487

[B44] Spector A. A. Yorek M. A. (1985). Membrane Lipid Composition and Cellular Function. J. Lipid Res. 26 (9), 1015–1035. 10.1016/s0022-2275(20)34276-0 3906008

[B45] Tang C. Wang Y. Lv H. Guan Z. Gu J. (2020). Caution against Corticosteroid-Based COVID-19 Treatment. Lancet 395 (10239), 1759–1760. 10.1016/s0140-6736(20)30749-2 32464115 PMC7247780

[B46] Tao Y. Chen X. Cai H. Li W. Cai B. Chai C. (2017). Untargeted Serum Metabolomics Reveals Fu-Zhu-Jiang-Tang Tablet and its Optimal Combination Improve an Impaired Glucose and Lipid Metabolism in Type II Diabetic Rats. J. Chromatogr. B Analyt Technol. Biomed. Life Sci. 1040, 222–232. 10.1016/j.jchromb.2016.11.012 27866845

[B47] Wang X. Yang B. Sun H. Zhang A. (2012). Pattern Recognition Approaches and Computational Systems Tools for Ultra Performance Liquid Chromatography-Mass Spectrometry-Based Comprehensive Metabolomic Profiling and Pathways Analysis of Biological Data Sets. Anal. Chem. 84 (1), 428–439. 10.1021/ac202828r 22132738

[B48] Wang X. Y. Ma T. L. Chen K. N. Pang Z. Y. Wang H. Huang J. M. (2021). Accumulation of LDL/ox-LDL in the Necrotic Region Participates in Osteonecrosis of the Femoral Head: a Pathological and *In Vitro* Study. Lipids Health Dis. 20 (1), 167. 10.1186/s12944-021-01601-x 34823555 PMC8620162

[B49] Weinstein R. S. Jilka R. L. Parfitt A. M. Manolagas S. C. (1998). Inhibition of Osteoblastogenesis and Promotion of Apoptosis of Osteoblasts and Osteocytes by Glucocorticoids. Potential Mechanisms of Their Deleterious Effects on Bone. J. Clin. Invest. 102 (2), 274–282. 10.1172/jci2799 9664068 PMC508885

[B50] Wójcik P. Žarković N. Gęgotek A. Skrzydlewska E. (2020). Involvement of Metabolic Lipid Mediators in the Regulation of Apoptosis. Biomolecules 10 (3), 402. 10.3390/biom10030402 PMC7175142 32150849

[B51] Wu X. Ma Y. Chen H. Hao Z. Su N. Li X. (2019). Lysophosphatidic Acid Induces Interleukin-6 and CXCL15 Secretion from MLO-Y4 Cells through Activation of the LPA1 Receptor and PKCθ Signaling Pathway. Int. Immunopharmacol 74, 105664. 10.1016/j.intimp.2019.05.049 31233937

[B52] Wu X. N. Ma Y. Y. Hao Z. C. Wang H. (2020). Research Progress on the Biological Regulatory Function of Lysophosphatidic Acid in Bone Tissue Cells. Hua Xi Kou Qiang Yi Xue Za Zhi 38 (3), 324–329. 10.7518/hxkq.2020.03.017 32573143 PMC7296378

[B53] Wu Z. Ji C. Li H. Qiu G. Gao C. Weng X. (2013). Elevated Level of Membrane Microparticles in the Disease of Steroid-Induced Vascular Osteonecrosis. J. Craniofac. Surg. 24 (4), 1252–1256. 10.1097/SCS.0b013e3182902dd3 23851782

[B54] Xing Y. (2002). “A Clinical Study on the Treatment of Avascular Necrosis of the Femoral Head by Acupoint Injection of Gushuling Injection,” in Traditional Chinese Medicine (Shenyang: Liaoning College of Traditional Chinese Medicine).

[B55] Xu H. Wang C. Liu C. Peng Z. Li J. Jin Y. (2021). Cotransplantation of Mesenchymal Stem Cells and Endothelial Progenitor Cells for Treating Steroid-Induced Osteonecrosis of the Femoral Head. Stem Cell Transl Med 10 (5), 781–796. 10.1002/sctm.20-0346 PMC8046137 33438370

[B56] Xu J. Gong H. Lu S. Deasey M. J. Cui Q. (2018). Animal Models of Steroid-Induced Osteonecrosis of the Femoral Head-A Comprehensive Research Review up to 2018. Int. Orthop. 42 (7), 1729–1737. 10.1007/s00264-018-3956-1 29705870

[B57] Xu X. Wen H. Hu Y. Yu H. Zhang Y. Chen C. (2014). STAT1-caspase 3 Pathway in the Apoptotic Process Associated with Steroid-Induced Necrosis of the Femoral Head. J. Mol. Histol. 45 (4), 473–485. 10.1007/s10735-014-9571-6 24554068

[B58] Yamamoto T. Irisa T. Sugioka Y. Sueishi K. (1997). Effects of Pulse Methylprednisolone on Bone and Marrow Tissues: Corticosteroid-Induced Osteonecrosis in Rabbits. Arthritis Rheum. 40 (11), 2055–2064. 10.1002/art.1780401119 9365096

[B59] Yan Z. Zhan J. Qi W. Lin J. Huang Y. Xue X. (2020). The Protective Effect of Luteolin in Glucocorticoid-Induced Osteonecrosis of the Femoral Head. Front. Pharmacol. 11, 1195. 10.3389/fphar.2020.01195 32903480 PMC7435053

[B60] Yin Z. Wang X. Yang X. Chen Y. Duan Y. Han J. (2021). Salvia Miltiorrhiza in Anti-diabetic Angiopathy. Cmp 14, 960–974. 10.2174/1874467214999210111222918 33430756

[B61] Yokoi N. Beppu M. Yoshida E. Hoshikawa R. Hidaka S. Matsubara T. (2015). Identification of Putative Biomarkers for Prediabetes by Metabolome Analysis of Rat Models of Type 2 Diabetes. Metabolomics 11 (5), 1277–1286. 10.1007/s11306-015-0784-9 26366137 PMC4559098

[B62] Yu X. Chen K. Lv X. Fan X. (2014). Study on Prevention of Gushiling Capsule on Hormone-Related Femoral Head Necrosis Based on Curing Before-Disease Theory of Traditional Chinese Medicine. Chin. J. Traditional Med. Sci. Tech. 21 (4), 3.

[B63] Zhang A. Sun H. Han Y. Yuan Y. Wang P. Song G. (2012). Exploratory Urinary Metabolic Biomarkers and Pathways Using UPLC-Q-TOF-HDMS Coupled with Pattern Recognition Approach. Analyst 137 (18), 4200–4208. 10.1039/c2an35780a 22852134

[B64] Zhang F. Peng W. Zhang J. Dong W. Wu J. Wang T. (2020). P53 and Parkin Co-regulate Mitophagy in Bone Marrow Mesenchymal Stem Cells to Promote the Repair of Early Steroid-Induced Osteonecrosis of the Femoral Head. Cell Death Dis 11 (1), 42. 10.1038/s41419-020-2238-1 31959744 PMC6971291

[B65] Zhang G. Xing L. (2016). Gushuling Combined with Minimally Invasive Surgery in the Treatment of ARCO Stage Ⅰ and Ⅱ Femoral Head Necrosis. Chin. J. Med. Guide 18 (12), 1270–1271+1273.

[B66] Zhang H. Y. Chen X. Hu P. Liang Q. L. Liang X. P. Wang Y. M. (2009). Metabolomic Profiling of Rat Serum Associated with Isoproterenol-Induced Myocardial Infarction Using Ultra-performance Liquid Chromatography/time-Of-Flight Mass Spectrometry and Multivariate Analysis. Talanta 79 (2), 254–259. 10.1016/j.talanta.2009.03.045 19559874

[B67] Zhang J. Wang Z. Hong G. (2021). Clinical Value of Digital Tomographic Fusion Imaging in the Diagnosis of Avascular Necrosis of the Femoral Head in Adults. Ir J. Med. Sci. 190 (4), 1585–1589. 10.1007/s11845-020-02451-9 33403520 PMC8521531

[B68] Zhang Y. F. Wei Y. S. Huang X. X. Zhu L. X. (2019). Brain lipidomics of intervention effect of Salviae Miltiorrhizae Radix et Rhizoma and Chuanxiong Rhizoma on ischemic stroke based on UPLC-Q-TOF-MS technique. Zhongguo Zhong Yao Za Zhi 44 (20), 4511–4518. 10.19540/j.cnki.cjcmm.20190321.404 31872640

[B69] Zhao T. Zhang H. Zhao T. Zhang X. Lu J. Yin T. (2012). Intrarenal Metabolomics Reveals the Association of Local Organic Toxins with the Progression of Diabetic Kidney Disease. J. Pharm. Biomed. Anal. 60, 32–43. 10.1016/j.jpba.2011.11.010 22153801

[B70] Zhou L. Wang Q. Yin P. Xing W. Wu Z. Chen S. (2012). Serum Metabolomics Reveals the Deregulation of Fatty Acids Metabolism in Hepatocellular Carcinoma and Chronic Liver Diseases. Anal. Bioanal. Chem. 403 (1), 203–213. 10.1007/s00216-012-5782-4 22349331

[B71] Zhu C. Liang Q. L. Hu P. Wang Y. M. Luo G. A. (2011). Phospholipidomic Identification of Potential Plasma Biomarkers Associated with Type 2 Diabetes Mellitus and Diabetic Nephropathy. Talanta 85 (4), 1711–1720. 10.1016/j.talanta.2011.05.036 21872008

