# Supplementary material for: Metabolic Profiling Analysis of the Effect and Mechanism of Gushiling Capsule in Rabbits With Glucocorticoid-Induced Osteonecrosis of the Femoral Head
Source: Front Pharmacol. 2022 May 2;13:845856. doi: 10.3389/fphar.2022.845856 (PMC9108178; doi:10.3389/fphar.2022.845856)
Supplement: Supplementary file 1 [file DataSheet1.docx]

Supplementary Material

# Supplementary Figures and Tables

## Supplementary Figures


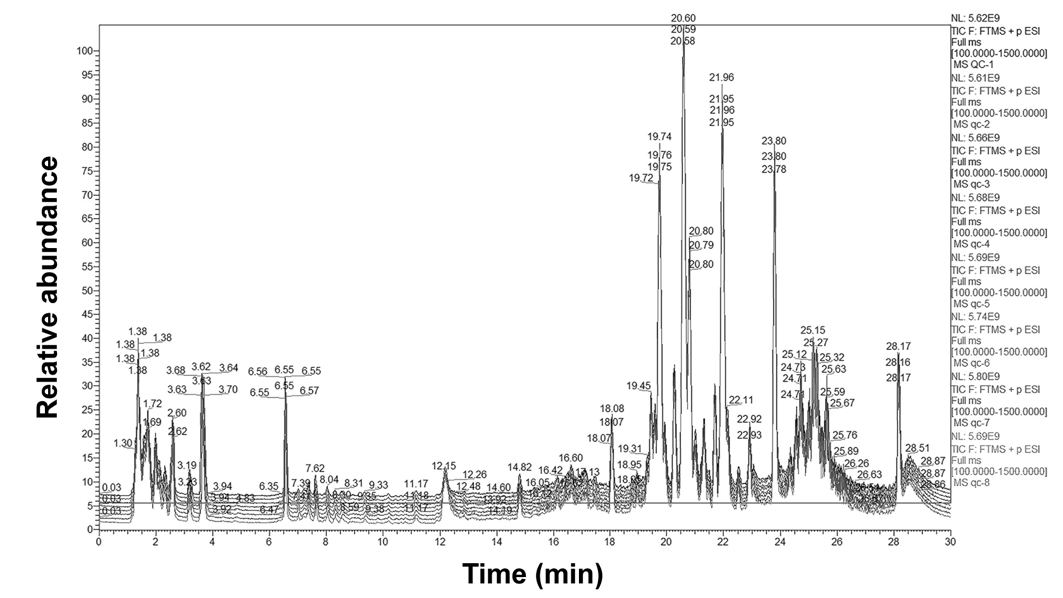


**Supplementary Figure 1** A typical LC-MS total ion current (TIC) chromatogram of rabbit serum samples in the Quality Control (QC) group.


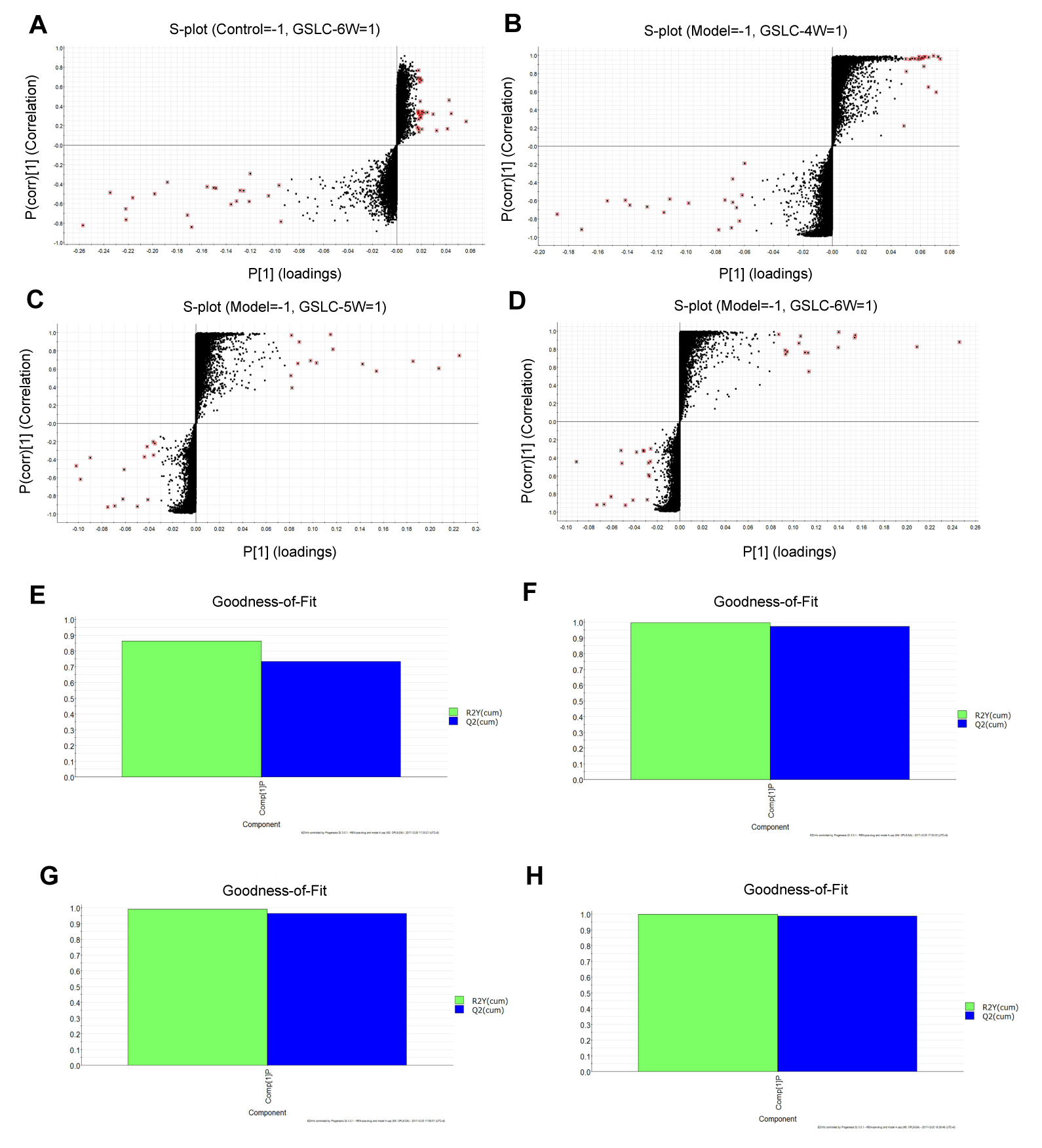


**Supplementary Figure 2** S-plot and Goodness-of-Fit for serum samples analysis in different stages. The S-plots of (**A**) the control group vs. GSLC group (6 weeks) and (**B–D**) the model group vs. GSLC groups (4, 5, and 6 weeks). (**E–H**) The Goodness-of-Fit of the control group vs. GSLC group (6 weeks) and the model group vs. GSLC groups at 4, 5 and 6 weeks, respectively.


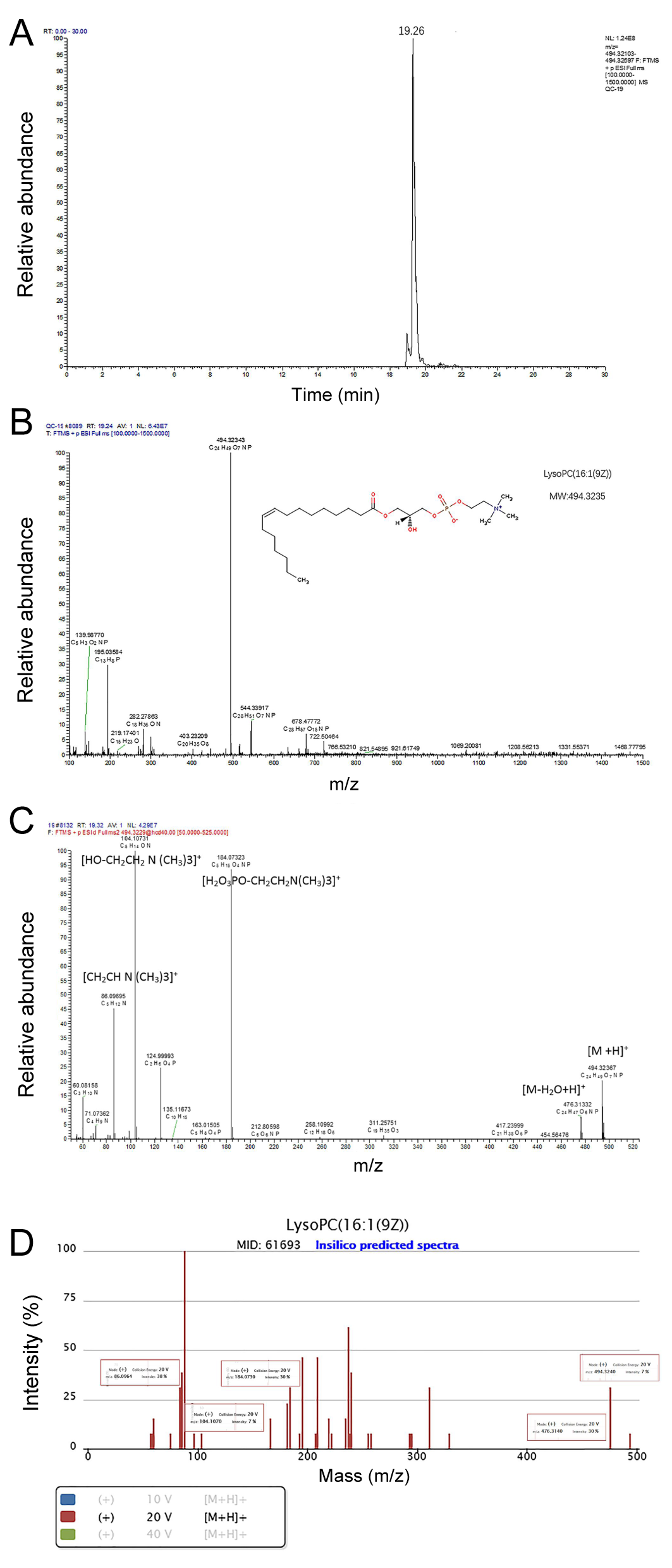


**Supplementary Figure 3.** Identification of a selected marker. (**A**) Peak of potential biomarker of m/z 494 in extracted ion chromatogram with positive mode; (**B**) The structure and corresponding mass spectrum of representative marker compound; (**C**) MS/MS spectrum on the collision energy was 20 eV; (**D**) Standard MS/MS fragmentation in Metlin databases.


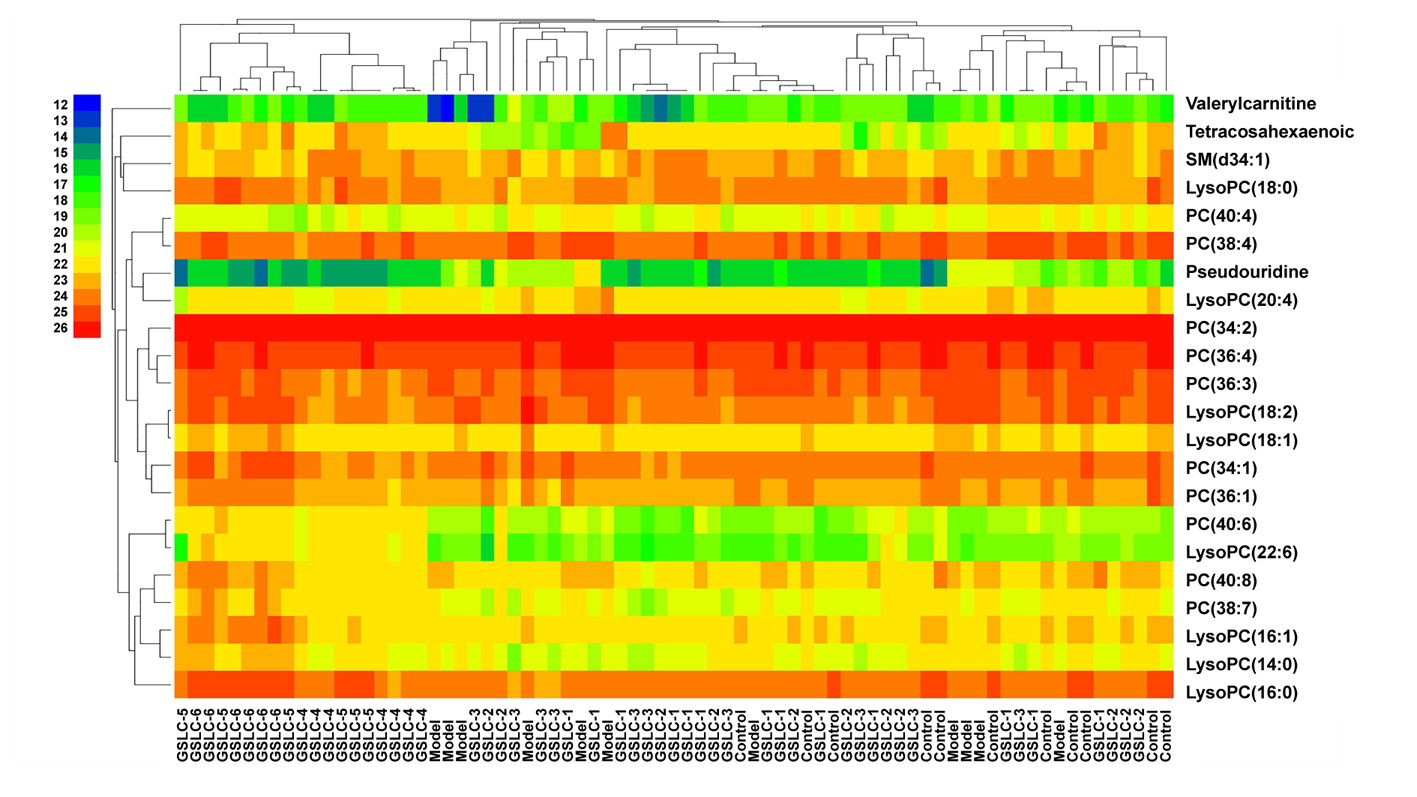


**Supplementary Figure 4.** Heatmap of the potential biomarkers from rabbit serum samples in different groups. Horizontal is the clustering of potential biomarkers; vertical is the clustering of different groups of samples. Red represents high-abundance biomarkers, blue represented low-abundance biomarkers.
